# Post-Pandemic Differences in Symptom Networks and Personality Associations Among College Students: A Repeated Cross-Sectional Study

**DOI:** 10.3390/bs16071171

**Published:** 2026-07-11

**Authors:** Yue Tang, Changyu Liu, Peng Lei, Huoyin Zhang, Xu Xu, Xuexuan Ning, Junyi Li, Wenshu Fan

**Affiliations:** 1 College of Psychology, Sichuan Normal University, Chengdu 610066, China; 2 School of Tourism and Cultural Industries, Sichuan Tourism University, Chengdu 610100, China; 3 Department of Military Psychology, Faculty of Medical Psychology, Army Medical University, Chongqing 400038, China; 4 Mental Health Education Center for College Students, Sichuan Normal University, Chengdu 610066, China; 5 Mental Health Education Center for College Students, Chengdu University of Traditional Chinese Medicine, Chengdu 610075, China

**Keywords:** mental health, college students, personality traits, COVID-19, network analysis

## Abstract

The COVID-19 pandemic has substantially affected college students’ mental health worldwide. Although daily life has gradually returned to normal, less is known about whether the underlying structure of psychological symptoms—and their associations with personality traits—has shifted in the post-pandemic period. Addressing this gap, the present study applied network analysis to psychological and personality data from 51,305 college freshmen to compare pre-pandemic and post-pandemic cohorts. Using the Eysenck Personality Questionnaire (EPQ) and the Symptom Checklist-90 (SCL-90), networks based on EPQ dimensions and SCL-90 subscale scores were estimated and compared across pre-pandemic and post-pandemic cohorts. Results indicated that anxiety and depression remained central symptom domains across both periods; however, the network-structure invariance test was significant. Specifically, the expected influence (EI) of both interpersonal sensitivity (ΔEI = 0.058, *p* = 0.007) and paranoia (ΔEI = 0.059, *p* = 0.007) showed a significant increase. The regularized partial associations between symptoms and personality traits also changed, with the negative correlation between extraversion and interpersonal sensitivity weakening, and the positive correlation between neuroticism and obsessive–compulsive symptoms strengthening. These findings suggest that although overall mental health showed no substantial deterioration compared to the pre-pandemic period, the underlying symptom network and its associations with personality traits differ between cohorts, highlighting the potential value of considering personality-related information in future research on post-pandemic mental health among university students.

## 1. Introduction

The COVID-19 pandemic, which broke out in 2019, was a global public health crisis that had a profound impact on the physical and mental health of the worldwide population (Duan & Zhu, 2020). Among the affected populations, college freshmen may have faced particularly severe challenges. On one hand, this group is at a critical stage of psychological and social development, during which they must adapt to significant changes in their social environment and support systems (Credé & Niehorster, 2012; Conley et al., 2013), while also being at increased risk for the onset of mental disorders (Chung & Hudziak, 2017). On the other hand, the implementation of quarantine policies and the shift to online learning heightened the demands on their self-regulation and time management, thereby further increasing the likelihood of adjustment difficulties (King et al., 2023). Compared to their senior counterparts, they experienced more pronounced declines in mental health during the pandemic, including more severe and persistent symptoms of anxiety, depression, and sleep disturbances (Fruehwirth et al., 2021; Turner et al., 2023). Therefore, it is imperative to systematically investigate the differences in college freshmen’s mental health before and after the pandemic, identify key symptoms, and explore potential protective factors, in order to provide empirical support for the development of personalized intervention strategies.

### 1.1. Background

#### 1.1.1. Mental Health Changes in the Post-Pandemic Period

On 8 January 2023, the Chinese government shifted COVID-19 management toward normalized prevention and control, marking a major policy transition in China’s pandemic response. However, trends in the mental health status of college students in the post-pandemic period remain controversial. Some scholars argue that the mental health status of college students continues to be a serious concern in the aftermath of the pandemic. The COVID-19 pandemic not only inflicted substantial psychological damage during its acute phase but also exerted prolonged psychological distress that persists well beyond the crisis period (He et al., 2025; Q. Zhang et al., 2025; D. Wang et al., 2022). However, several longitudinal studies and meta-analyses have found that mental health outcomes have shown signs of improvement following the lifting of lockdown measures and the global decline of the pandemic (Qiao et al., 2023; Reutter et al., 2024). These inconsistencies in previous research findings may be attributed to several factors. On the one hand, differences in measurement instruments, sampling characteristics, study design, and the historical periods in which data were collected may contribute to variability across studies. On the other hand, the pandemic represents a prolonged and multifaceted societal stressor, and individuals may exhibit markedly different psychological responses to the same stressor due to heterogeneity in personal characteristics and exposure to diverse environmental stressors. Therefore, the psychological impact of the pandemic is likely to operate through more complex and dynamic mechanisms than can be captured by any single study design or sample.

#### 1.1.2. Personality Trait and Mental Health

Moreover, personality traits, as a key component of individual diathesis, may also play a critical role in the variation in mental health. A large body of research in psychology has shown that personality traits have been consistently linked to mental health outcomes (Kang et al., 2023). The Eysenck Personality Model is one of the most commonly used models for classifying personality dimensions, including Extraversion, Neuroticism, and Psychoticism (Eysenck & Eysenck, 1975). The Extraversion dimension reflects the degree to which an individual is outgoing, sociable, and active in social interactions. The Neuroticism dimension pertains to emotional stability, with higher scores indicating greater emotional lability and a tendency to experience anxiety, worry, and depressive mood. The Psychoticism dimension is associated with traits such as stubbornness, social isolation, and difficulty in adapting to others (Eysenck & Eysenck, 1975). Currently, there is still controversy surrounding the relationship between personality and mental health in the context of the pandemic. Some studies have found that psychological symptom severity is positively associated with neuroticism and psychoticism, and negatively associated with extraversion (Liao et al., 2017; Pauly et al., 2021; L. Wang et al., 2023). However, some studies have found that individuals with high levels of extraversion experienced more psychological difficulties during the pandemic (Staneva et al., 2022), whereas those with lower levels of extraversion appeared to adapt more easily to pandemic-related lifestyle changes (Choi et al., 2022).

### 1.2. Limitations of Existing Approaches

This contradictory pattern of findings suggests that additional analytical perspectives may help provide a more comprehensive understanding of the relationship between personality and psychological symptoms. Traditional latent-variable methods view observed symptoms as indicators of underlying latent constructs (Bollen, 2002), providing a parsimonious framework for understanding broad psychological dimensions. However, these methods may not fully characterize the conditional associations among symptom domains and may overlook the complex patterns of relationships that exist across symptoms (Borsboom & Cramer, 2013; Borsboom, 2017).

In contrast, network analysis can identify relatively central symptom domains within a symptom network (Borsboom & Cramer, 2013). Rather than conceptualizing psychological disorders solely as manifestations of latent constructs, this approach represents symptoms as interconnected components linked by conditional associations, allowing researchers to characterize the structure of relationships among symptom domains (van Borkulo et al., 2023). Cramer et al. (2012) further suggested that, in personality research, network analysis provides a complementary perspective to traditional latent-variable models. Their work demonstrated that network analysis can also be applied to personality dimensions. In the context of post-pandemic mental health research, this approach may help clarify how personality traits are associated with the psychological symptom domains, thereby providing a useful framework for examining cohort differences before and after the pandemic.

### 1.3. Theoretical Framework

To more systematically examine the differences in college freshmen’s mental health in the post-pandemic period, this study adopts the diathesis–stress theory (Monroe & Simons, 1991) as its core theoretical framework. This theory has been widely applied to research on the psychological impact of COVID-19 among university students (Moeller et al., 2022; Hong et al., 2021; C. Yang, 2024), demonstrating both strong explanatory power and practical value. According to the diathesis–stress theory, psychological problems or mental disorders are the result of the interaction between diathesis and stress (Monroe & Simons, 1991). Diathesis refers to an individual’s physiological, psychological, or environmental vulnerabilities or susceptibilities, which are influenced by factors such as genetics, neural activity, personality traits, and early life experiences. Environmental stress refers to the various negative events or environmental pressures an individual experiences in life, such as academic stress, work stress, interpersonal conflicts, economic difficulties, or major life changes. Compared with research approaches that focus solely on the direct impact of the pandemic, the diathesis–stress theory considers both internal vulnerabilities and external stressors, offering a useful framework for understanding college students’ mental health in the context of COVID-19 and for examining associations between personality traits and psychological symptoms across pre-pandemic and post-pandemic cohorts.

### 1.4. The Present Study

To address the above gaps, the present study applies a network analysis approach to large-scale repeated cross-sectional data to examine differences in college freshmen’s symptom severity and its association with personality traits before and after the COVID-19 pandemic.

This study makes three main contributions. First, it applies network analysis to examine symptom organization and centrality patterns beyond mean-level symptom comparisons. Second, it integrates personality traits into a diathesis–stress framework, providing a theoretically informed perspective on personality–symptom associations. Third, using a large-scale repeated cross-sectional design, it provides empirical evidence on post-pandemic mental health patterns among college freshmen.

Specifically, the study aims to: (1) compare differences in mean symptom dimension scores and total SCL-90 scores between the pre-pandemic and post-pandemic periods; (2) estimate and compare network structures, including edge weights, centrality indices, global network strength, and network invariance; and (3) explore the relationship between personality traits and symptom severity, as well as how this relationship differences across periods.

Based on the diathesis–stress framework, the following hypotheses are proposed:

**H1.**  *Compared with the pre-pandemic period, the mental health level of college freshmen has declined in the post-pandemic period.*

**H2.**  *Compared with the pre-pandemic period, the central symptom domains of college freshmen’s mental health have shifted in the post-pandemic period.*

**H3.**  *Symptom severity is positively associated with neuroticism and psychoticism, and negatively associated with extraversion.*

## 2. Methods

### 2.1. Participants

**Figure 1:**
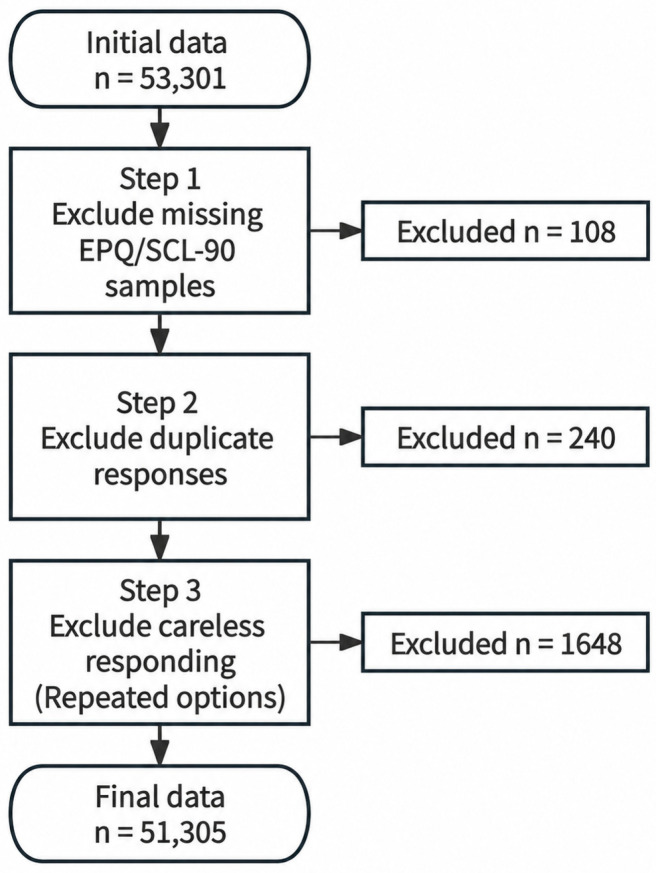
Data Processing Flowchart.

The data used in this study were collected from the mental health and personality assessment data of freshmen enrolled at a comprehensive university in Chengdu, Sichuan Province, China, which admits students through the national unified college entrance examination, both before (2017–2019) and after (2023–2024) the pandemic. The assessment procedure and administration protocol were identical across all study years. Data collection was conducted within the first two months after enrollment (September–October each year) as part of the university’s routine psychological health screening. According to university policy, all incoming students are required to complete mental health assessments as part of routine psychological screening and support services. Data were collected via online survey questionnaires, and all participants signed an electronic informed consent form that included consent for the use of anonymized data for future research purposes. No financial or material incentives were provided. A total of 53,301 responses were collected, with 32,517 from the pre-pandemic period (T1) and 20,784 from the post-pandemic period (T2). Data cleaning proceeded in three steps. First, 108 responses without complete SCL-90 or EPQ data were removed. Second, 240 duplicate responses from the same participant were identified and excluded, retaining only the first completed response. Third, responses indicative of careless responding—operationalized as selecting the same response option for every item on either the SCL-90 or the EPQ—were excluded (n = 1648). After these exclusions, the final valid sample comprised 51,305 responses (validity rate: 96.26%), of which 31,726 were from T1 and 19,579 from T2. The data processing flowchart is presented in Figure 1. This retrospective study was conducted in accordance with the Declaration of Helsinki. Retrospective ethics approval was obtained from the Ethics Review Sub-committee of the Sichuan Psychological Society of China (No. 2024029) on 18 July 2024. No participants under 18 years of age were included in the dataset used for analysis.

### 2.2. Measurement Tools

#### 2.2.1. Eysenck Personality Questionnaire

The Chinese revised version of the Eysenck Personality Questionnaire (EPQ), developed by Gong (1984), was used to assess personality traits. To reduce participant burden during the large-scale campus mental health screening, the Lie (L) scale was omitted. Accordingly, the version used in the present study included the Extraversion (E), Neuroticism (N), and Psychoticism (P) subscales only. The questionnaire consisted of 68 dichotomous (Yes/No) items. Standardized T-scores provided in the dataset were used in the present analysis, with higher T-scores indicating higher levels of the corresponding personality trait. Cronbach’s α coefficients for the Extraversion, Neuroticism, and Psychoticism subscales were 0.821, 0.880, and 0.579 in T1, and 0.843, 0.900, and 0.616 in T2, respectively. Although the Cronbach’s α for the Psychoticism scale was relatively low in both periods, this is consistent with prior findings, as the Psychoticism subscale has consistently been reported to yield lower internal consistency compared to the other EPQ subscales (Caruso et al., 2001; Jiao et al., 2011).

#### 2.2.2. Symptom Checklist-90

Psychological symptoms were assessed using the Chinese version of the Symptom Checklist-90 (SCL-90), originally translated and adapted by Z. Wang (1984) and subsequently revalidated by Chen and Li (2003). The SCL-90 contains 90 items assessing nine symptom dimensions: Somatization (SOM), Obsessive–compulsive (OC), Interpersonal sensitivity (IS), Depression (DEP), Anxiety (ANX), Hostility (HOS), Phobic anxiety (PHOB), Paranoia (PAR), and Psychoticism (PSY). Each item is rated on a 5-point Likert scale ranging from 1 (“not at all”) to 5 (“extremely”). Factor scores were calculated as the arithmetic mean of the items comprising each dimension, with higher scores indicating greater symptom severity. Cronbach’s α coefficients for the nine dimensions ranged from 0.760 to 0.907 in T1 and from 0.821 to 0.925 in T2.

#### 2.2.3. Demographic Variables

A demographic questionnaire was designed to collect information on gender, hometown (urban vs. rural), only-child status (yes vs. no), divorced family background (parents divorced vs. not), foster care experience (raised primarily by non-parental caregivers vs. not), and left-behind experience. Left-behind experience was assessed using a screening question with five options: no left-behind experience, left behind and lived with mother, left behind and lived with father, left behind and lived with grandparents, and left behind and lived with others (Murphy et al., 2016); participants who reported any form of left-behind experience were classified into the “with left-behind experience” group. Childhood trauma was assessed using the Childhood Trauma Screener (Grabe et al., 2012), which evaluates five types of traumatic experiences: physical neglect, physical abuse, sexual abuse, emotional abuse, and emotional neglect. Participants who reported experiencing any one or more of these five types were classified as the “with childhood trauma” group.

### 2.3. Data Analysis

Descriptive statistical analyses were conducted using SPSS 26.0 to characterize the sample and examine the distributional properties of the study variables. Means, standard deviations, skewness, and kurtosis were calculated for all 12 network nodes within each cohort. Between-cohort comparisons were then performed, including (a) independent-samples t-tests for all node means with Holm–Bonferroni correction for multiple comparisons, (b) chi-square tests for categorical demographic variables, and (c) the calculation of standardized effect sizes (Cohen’s *d* and Cramer’s *V*) to quantify the magnitude of group differences. In addition, three separate sets of independent-samples t-tests were conducted to compare node scores across gender, only-child status, and childhood trauma experience. Holm–Bonferroni correction was likewise applied to these analyses, and Cohen’s d was reported to facilitate interpretation of the magnitude of group differences (Supplementary Tables S1–S3). Network analysis was conducted using R 4.5.0, and the analysis followed the reporting standards for psychological network analysis outlined by Burger et al. (2023). This study constructed a network with 12 nodes, using the three dimensions of the EPQ and the nine factors of the SCL-90 as variables. To ensure the uniqueness of the nodes, redundancy detection was performed prior to the network estimation. Node redundancy was assessed using the goldbricker() function from the R package networktools (version 1.5.2), with default arguments (corMin = 0.5, threshold = 0.25). No node pairs met the redundancy criteria, confirming that all 12 nodes were sufficiently distinct and retained in the network.

#### 2.3.1. Estimation Method

Given that our data consists of continuous variables, we estimated the partial correlation network using the Gaussian Graphical Model (GGM). Sparse estimation was performed using the Least Absolute Shrinkage and Selection Operator (LASSO), and model selection was carried out using the Extended Bayesian Information Criterion (EBIC) to minimize the false-positive edges in the network (Fried et al., 2018). The tuning parameter γ was set to 0.5 to construct the sparse partial correlation network. Specifically, network estimation was carried out using the estimateNetwork function (setting default = “EBICglasso” and threshold = TRUE, with Spearman’s rho correlations computed via pairwise.complete.obs) in the R package bootnet 1.6 (Epskamp et al., 2018). To facilitate visual comparison between the two networks, both networks were plotted using a common layout generated by the averageLayout() function in the package qgraph 1.9.8 (Epskamp et al., 2012), in which node positions were determined based on the average of the two network structures and held constant across panels.

#### 2.3.2. Centrality Indices and Predictability

The node predictability was calculated using the R package MGM 1.2-14. Specifically, the R^2^ of each node was used as a key metric (Haslbeck & Waldorp, 2020), representing the proportion of variance in a given node that can be explained by its neighboring nodes in the estimated network. From a network perspective, higher predictability indicates that a larger proportion of a node’s variance is accounted for by the variables included in the network, whereas lower predictability suggests that a greater proportion of its variance may be attributable to factors not included in the current model. The centrality index aims to identify relatively important nodes in the network, with symptoms having high centrality often being closely linked to other symptoms in the network (Robinaugh et al., 2016). In this study, Expected Influence (EI) was used as the primary centrality index to quantify node importance within the network. EI is computed by summing all edge weights directly connected to a node while retaining their positive and negative signs, making it particularly suitable for networks containing negative associations (Robinaugh et al., 2016). Drawing on the top-percentile criterion previously used to identify core bridge symptoms, we adopted a 20% threshold to define central symptom domains in the present study (Robinaugh et al., 2016).

#### 2.3.3. Stability and Accuracy Estimation

To assess the accuracy of edge estimates, a non-parametric percentile bootstrap procedure implemented in the R package bootnet 1.6 (Epskamp et al., 2018) was used to estimate the 95% percentile confidence intervals for edge weights (1000 bootstrap samples). Narrower confidence intervals indicate greater accuracy of the estimated edge weights. Additionally, the case-dropping bootstrap (1000 bootstrap samples) method was used to delete a certain proportion of samples to determine whether the ranking of node centrality indices would change. The stability of the centrality indices was measured by the correlation stability coefficient (CS-coefficient), which assesses the maximum proportion of samples that can be deleted at a 95% confidence level while maintaining a correlation between the centrality index and the original estimate of at least 0.7. The CS-coefficient should not be lower than 0.25, with an optimal value above 0.5.

#### 2.3.4. Group Comparison

We compared differences between the pre-pandemic and post-pandemic networks from both global and local perspectives. Global comparisons included tests of network structure invariance and global strength, whereas local comparisons included tests of edge-weight differences and node expected influence differences. This comparison was conducted using the R package NetworkComparisonTest 2.2.2 (van Borkulo et al., 2023), employing a permutation test with 5000 iterations. The test was performed on regularized networks estimated with γ = 0.5 based on Spearman correlation matrices. Differences in all edge weights (test.edges = TRUE) and node expected influence (test.centrality = TRUE, centrality = “expectedInfluence”) were evaluated, with the Holm–Bonferroni method applied for multiple comparison correction. The NCT() function was run with binary.data = FALSE, nodes = “all”, and paired = FALSE, as the two samples were independent. In addition, to assess within-period heterogeneity, network comparison tests were conducted between individual academic years within each period.

## 3. Results

### 3.1. Descriptive Statistics and Demographic Information

The means, standard deviations, skewness and kurtosis of the pre-pandemic and post-pandemic data are shown in Table 1. Although most symptom dimension scores and the total SCL-90 score differed significantly between the two periods, all effect sizes were small, indicating that the overall differences between the two cohorts were limited. In addition, although all demographic characteristics differed statistically significantly between T1 and T2, the corresponding effect sizes were small (all Cramér’s *V* < 0.10 except for only-child status), suggesting limited practical differences between samples. Detailed demographic information is shown in Table 2.

### 3.2. Network Analysis

#### 3.2.1. Network Visualization, Edge Weight, and Density

**Figure 2:**
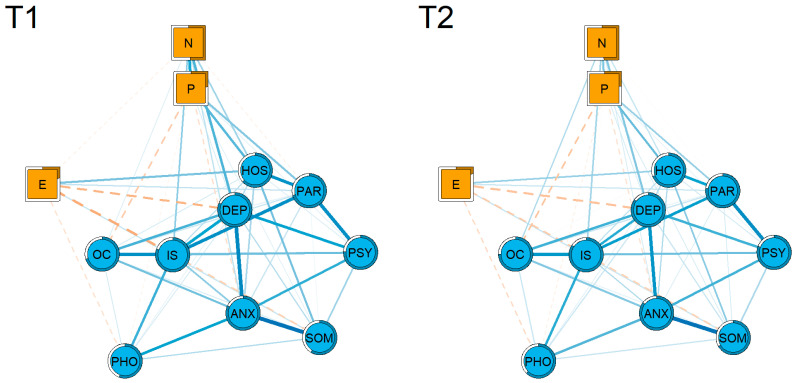
Gaussian graphical models of symptom domains and personality traits. Note: Blue circular nodes represent psychological symptom dimensions, whereas orange square nodes represent personality trait dimensions. Edges represent regularized partial correlations estimated using the EBICglasso procedure. Blue solid edges indicate positive partial correlations, whereas orange dashed edges indicate negative partial correlations. Edge thickness is proportional to the absolute magnitude of the regularized partial correlation. To facilitate visual comparison between the two networks, both networks were plotted using a common average layout, in which node positions were determined from the average of the two network structures and then held constant across panels. T1 refers to the pre-pandemic period, and T2 refers to the post-pandemic period. The rings outside the nodes represent the predictability values.

The redundancy check showed that there was no node redundancy, meaning each node is unique. Figure 2 shows the networks at T1 and T2 periods. Each node in the figure represents a symptom domain or personality trait, with edges between nodes representing regularized partial correlations, and the rings outside the nodes representing the predictability values. The network at T1 consists of 57 edges, with a network density of 86.36%. The edge weights range from −0.158 (Extraversion–Interpersonal Sensitivity) to 0.293 (Anxiety–Somatization), with an average edge weight of 0.070 (the complete edge weight matrix is shown in Supplementary Table S4). The network at T2 consists of 56 edges, with a network density of 84.85%. The edge weights range from −0.131 (Extraversion–Depression) to 0.329 (Anxiety–Somatization), with an average edge weight of 0.071 (the complete edge weight matrix is shown in Supplementary Table S5). Notably, the estimated networks exhibited relatively high density, a pattern that is broadly consistent with previous SCL-90-based network studies reporting widespread associations among symptom domains (W.-X. Zhang et al., 2024; Arrindell et al., 2026). This pattern may be attributable to two factors. First, the large sample size allows relatively small but stable partial correlations to be estimated. Second, the broad psychological constructs measured by the SCL-90 and EPQ are theoretically expected to exhibit widespread conditional associations across domains.

#### 3.2.2. Centrality Indices and Predictability

**Figure 3:**
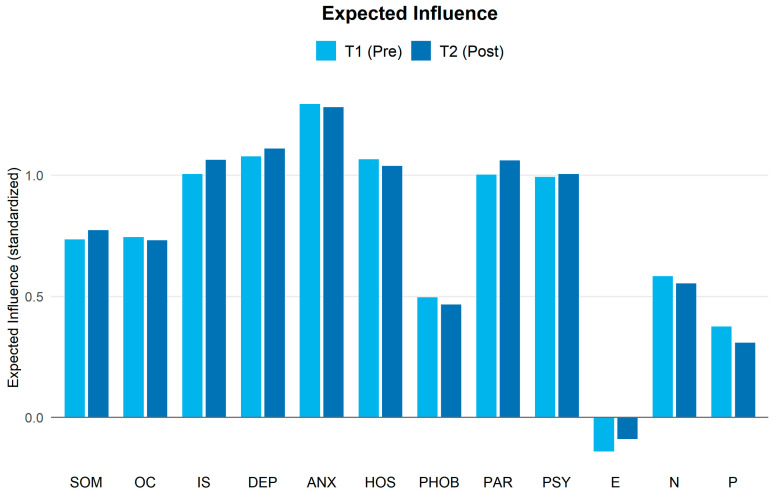
Expected influence values of the two periods. Note: Expected influence values are presented as raw (unstandardized) estimates. T1 refers to the pre-pandemic period, and T2 refers to the post-pandemic period.

Figure 3 presents the results of the centrality analysis for the two periods. Additionally, Supplementary Table S6 lists the original EI, and the predictability results are presented in Supplementary Table S7. Across both T1 and T2, anxiety and depression consistently emerged as the two most central symptom domains (see Supplementary Table S6 for specific values), suggesting their stable core positions within the network.

#### 3.2.3. Results of the Accuracy and Stability Checks

The bootstrapping results for edge weights (see Supplementary Figure S1) showed relatively narrow confidence intervals, indicating acceptable accuracy of edge estimates. The stability analysis for centrality indices (see Supplementary Figure S2) was reported separately. The case-dropping bootstrap yielded a CS-coefficient of 0.75, indicating good stability of centrality indices.

#### 3.2.4. Group Comparisons

From a global network perspective, there were no significant differences in network strength between the two periods (GS(T1) = 6.024, GS(T2) = 6.031, S = 0.008, *p* = 0.863). However, significant differences were observed in the network structure (M = 0.050, *p* < 0.001). To further examine the structural differences between the networks, we compared node centrality and edge weights across the two time periods. The results revealed significant differences in EI for the nodes “Interpersonal Sensitivity” and “Paranoia.” Specifically, the EI for both “Interpersonal Sensitivity” and “Paranoia” were significantly higher in T2 compared to T1. Regarding edge differences, six edges exhibited significant variations between the two periods (see Supplementary Table S8). This study focuses on changes in the relationships between symptoms and personality traits. Among the edges meeting this criterion, two showed significant differences: “Extraversion–Interpersonal Sensitivity” and “Neuroticism–Obsessive–compulsive”. Within-period heterogeneity analyses indicated the presence of differences across academic years. Within T1 (2017–2019), significant differences in network structure were observed between 2017 and 2018 and between 2018 and 2019, while no significant differences in global strength were found. Within T2 (2023–2024), significant differences were observed in both network structure and global strength. Detailed results are provided in Supplementary Table S9.

## 4. Discussion

This study examined differences in mental health and personality-related networks among college freshmen before and after the pandemic. The results indicate that the most central domains in the networks mainly included “Anxiety” and “Depression”. Although no significant difference was observed in global network strength between the two periods, the estimated network structures differed significantly. Specific edge-weight comparisons showed that the connection between extraversion and interpersonal sensitivity was weaker in the post-pandemic cohort, whereas the association between obsessive–compulsive symptoms and neuroticism was stronger. Furthermore, the EI of both “Interpersonal Sensitivity” and “Paranoia” significantly increased after the pandemic.

### 4.1. Limited Differences in Symptom Levels Between T1 and T2 Cohorts

Direct comparisons of the SCL-90 total score and symptom dimension scores revealed statistically significant differences between the pre-pandemic and post-pandemic cohorts. However, the corresponding effect sizes were uniformly small, indicating that overall psychological symptom levels were largely comparable across the two periods, contrary to H1. This pattern suggests that overall symptom levels did not differ substantially between cohorts, despite differences in their pandemic-related experiences prior to entering university. One possible hypothesis is that the gradual resumption of in-person teaching and campus activities in the post-pandemic period may have helped restore opportunities for peer interaction and social connection, which previous studies have identified as important factors related to college students’ mental health (Qiao et al., 2023; Reutter et al., 2024). However, because the present study did not directly measure teaching arrangements, campus activities, social support, or peer interactions, this explanation should be interpreted cautiously and requires further empirical examination.

### 4.2. The Relationship Between Psychological Symptoms and Personality Traits and Their Changes

The results showed that the expected influence of extraversion was negative in both time periods, whereas the expected influence values of psychoticism and neuroticism were positive. Because expected influence is calculated as the sum of all edge weights connected to a node, these values should be interpreted as reflecting the net direction and magnitude of each node’s associations within the estimated network, rather than as direct evidence of a global positive or negative relationship with mental health. When considered together with the scoring direction and the specific edges involving personality and symptom nodes, this pattern suggests that extraversion was more strongly connected through negative edges, whereas neuroticism and psychoticism were more strongly connected through positive edges. This pattern is broadly consistent with H3 and aligns with findings from previous studies (Pauly et al., 2021; L. Wang et al., 2023). According to the diathesis–stress model, individuals with higher levels of extraversion generally demonstrate better social adaptability. Within this theoretical framework, extraversion has been proposed to function as a protective personality characteristic that may help individuals cope more effectively with stressful events (Soto, 2019). In contrast, higher levels of psychoticism have been associated with lower empathy and poorer impulse control (Huang et al., 2010), whereas higher neuroticism has consistently been associated with anxiety, depression, and emotional instability (Widiger & Oltmanns, 2017). Previous studies have also suggested that these personality characteristics may be associated with poorer social adaptation and greater psychological distress (Yan et al., 2014). Although the present study did not directly examine these mechanisms, our findings are broadly consistent with the diathesis–stress framework, which provides one possible perspective for interpreting the observed associations between personality traits and psychological symptoms.

The findings indicate that there are significant differences in the network structure of psychological symptoms before and after the pandemic. These differences were partly reflected in the changes in the strength of associations between symptoms and personality traits: the positive correlation between obsessive–compulsive symptoms and neuroticism increased post-pandemic, while the negative correlation between extraversion and interpersonal sensitivity weakened. First, from the perspective of the diathesis–stress model, the relationship between obsessive–compulsive symptoms and neuroticism may be interpreted as reflecting the joint influence of psychological vulnerability and environmental stressors. During pandemics, uncertainty about the future increases markedly. Previous studies have suggested that higher neuroticism is associated with more severe obsessive–compulsive symptoms, potentially through intolerance of uncertainty (Baerg & Bruchmann, 2022). Moreover, lockdown policies and online misinformation may intensify fear and uncertainty related to the pandemic, which may have been associated with stronger obsessive–compulsive symptoms among individuals with higher levels of neuroticism. The weakened negative association between extraversion and interpersonal sensitivity may reflect reduced social opportunities during lockdowns. This interpretation is consistent with previous findings suggesting that social deprivation may have limited opportunities for extraverted individuals to satisfy their interpersonal needs, potentially increasing discomfort and sensitivity in social interactions (Kohút et al., 2021). Longitudinal evidence suggests that pandemic-related social disruption may have enduring effects on social sensitivity, as social anxiety continued to rise above baseline levels years after the pandemic onset (Brown et al., 2024) and post-lockdown college students showed marginally higher social sensitivity compared to pre-lockdown cohorts (Cerutti et al., 2024).

### 4.3. Symptoms and Their Changes

Although some studies have suggested that the central symptom domains within psychological symptom networks may shift before and after the pandemic (Whang & Kim, 2026), the present study found that anxiety and depression remained stably central in the network across both periods, thereby failing to support H2. This finding suggests that, despite potential adjustments in the overall structure of psychological symptom networks under major contextual changes, affective symptoms such as depression and anxiety exhibit substantial stability. Existing literature provides partial support for this result. Multiple network analytic studies have consistently identified depression and anxiety as central symptom domains across different samples, including Chinese college students (Fan et al., 2025), adolescents (M. Yang et al., 2024; W.-X. Zhang et al., 2024), and children and adolescents across developmental stages (McElroy et al., 2018). On the other hand, a substantial body of research based on college student samples indicates that depression and anxiety are the most prevalent emotional problems and show high comorbidity (Li et al., 2022; Chang et al., 2021; McKenney et al., 2025; Zhou et al., 2020; Ma et al., 2020). The convergence of findings across populations and methodological approaches strengthens the robustness of the present results. These findings suggest that, even as pandemic-related stressors subside, these fundamental affective symptoms may continue to occupy central positions within psychological networks. Sustained attention to central symptom domains such as anxiety and depression may therefore be warranted in university mental health practice.

Another aspect of the structural differences in the networks lies in the changes in node centrality indices: in the post-pandemic period, the EI of both interpersonal sensitivity and paranoia increased significantly. Although interpersonal sensitivity and paranoia belong to different dimensions of the SCL-90, both have been linked to social information processing. Increasing evidence suggests that paranoia in the general population is not an isolated psychopathological phenomenon but rather a cognitive disposition closely associated with social threat processing and perceptions of interpersonal safety (Bell & O’Driscoll, 2018; Greenburgh et al., 2022). Paranoia has been proposed as one psychological process relevant to social functioning and is closely associated with social isolation, social withdrawal, and impaired social functioning (Cviková et al., 2026). Similarly, interpersonal sensitivity has been conceptualized as an interpersonal cognitive vulnerability characterized by excessive concern about social evaluation and interpersonal rejection. It is associated not only with emotional problems such as anxiety and depression but also with poorer interpersonal adjustment and reduced social functioning (Boyce & Parker, 1989; Starr et al., 2014). Taken together, interpersonal sensitivity and paranoia may represent distinct manifestations of social threat processing in interpersonal contexts and may reflect heightened sensitivity to potentially negative social cues.

In the context of the present study, the increased EI of interpersonal sensitivity and paranoia is more likely to reflect changes in the organization of the symptom network potentially associated with pandemic-related developmental experiences and the challenges associated with the transition to university, rather than the influence of any single factor. Compared with the pre-pandemic cohort, students entering university after the pandemic experienced a longer period of social restrictions, online learning, and reduced face-to-face peer interactions during late adolescence. Adolescence represents a critical developmental period for social cognition, peer relationships, and social functioning. Sustained peer interactions during this stage play an important role in establishing interpersonal trust, fostering a sense of social belonging, and shaping patterns of social information processing (Do et al., 2024; Blakemore, 2008; Kilford et al., 2016). Importantly, these pandemic-related developmental experiences may not have directly increased the severity of interpersonal sensitivity or paranoia; rather, they are more likely to have altered the way individuals process social information. Taylor (2022) proposed that the prolonged uncertainty associated with the COVID-19 pandemic, together with changes in patterns of interpersonal interaction, has been suggested to be associated with increased attention to social evaluation, others’ intentions, and potential interpersonal threats. The transition to university is accompanied by substantial interpersonal adjustment demands, with a sense of social belonging and the quality of peer relationships serving as key determinants of first-year students’ mental health (Credé & Niehorster, 2012; Conley et al., 2013). For students entering university after the pandemic, these developmental tasks unfolded against the backdrop of prolonged social restrictions and reduced peer interactions experienced during adolescence. Therefore, pandemic-related developmental experiences may not have directly increased the severity of interpersonal sensitivity or paranoid symptoms. Instead, they may have rendered these two forms of interpersonal cognition, both closely related to social threat processing, more centrally integrated within the overall symptom network and more strongly connected with symptoms such as anxiety, depression, and hostility, thereby contributing to their greater EI.

### 4.4. Strengths, Implications, and Limitations

Building on a large-scale dataset, a network analytical approach, and a theoretically informed diathesis–stress framework, this study provides an empirical examination of post-pandemic changes in college freshmen’s mental health and its association with personality traits. The findings have several potential implications for research and practice. First, from a practical and educational perspective, symptoms such as interpersonal sensitivity and paranoia, which showed increased centrality in the post-pandemic network, may warrant closer monitoring within university mental health services. Second, from a methodological perspective, incorporating network analysis into psychological assessment may provide complementary information beyond traditional total score–based approaches, allowing a more fine-grained understanding of associations among symptom domains. In particular, identifying highly central symptom domains, such as depression and anxiety in the present study, may help inform future research and monitoring efforts. Finally, personality traits may provide additional information for understanding individual differences in psychological symptom patterns; however, their potential role in risk stratification and applied assessment requires further investigation.

Despite these contributions, several theoretical and methodological limitations should be acknowledged, which future research may further address and improve upon.

First, and most importantly, the repeated cross-sectional design precludes causal inference. Observed between-cohort differences may reflect demographic composition changes, shifts in admission policies, societal trends, or unmeasured confounds rather than pandemic effects. The absence of individually measured pandemic-exposure variables is a critical limitation. Future research could incorporate a broader range of demographic and contextual variables and further examine their potential moderating effects on network structure. Second, the data from multiple academic years were pooled within each period; however, the within-period heterogeneity analyses indicated additional year-to-year differences, which may have obscured or introduced some bias into the pooled network estimates. Third, the diathesis–stress framework was only partially operationalised, as stress was represented at the cohort level rather than through individual-level measures of pandemic-related exposure. Therefore, variability in actual exposure experiences across individuals could not be directly assessed. Fourth, although the same instruments were used across time periods, differences in temporal context and administration conditions may have introduced potential measurement-related bias, and self-report questionnaires are also subject to response bias, which could not be fully ruled out. Fifth, differences in sample size and composition between the two cohorts may affect the stability and comparability of network estimation results, and should be considered when interpreting the findings. The study is also based on a single university sample, which may limit generalisability. Finally, although the centrality hypothesis suggests that targeting highly central symptom domains may facilitate overall symptom reduction, evidence indicates that the relationship between centrality indices in cross-sectional networks and intervention effects is not always stable or consistent (Bringmann et al., 2019; Dablander & Hinne, 2019; McNally, 2021). Future research should adopt longitudinal designs and clinical intervention studies to further examine and validate the robustness and causal relevance of the present findings.

## 5. Conclusions

This study employed network analysis to examine the relationship between mental health and personality traits among college freshmen in the post-pandemic period, with a comparative focus on networks before and after the COVID-19 pandemic. The findings showed that although differences in overall psychological symptom levels between the pre- and post-pandemic cohorts were small, and no significant difference was observed in global network strength, the estimated network structures differed. Notably, changes were observed in the associations between specific symptoms: the negative association between extraversion and interpersonal sensitivity decreased after the pandemic, while the positive association between neuroticism and obsessive–compulsive symptoms increased. Analysis of central symptom domains indicated that anxiety and depression occupied central positions in both networks, suggesting their strong correlation with other psychological symptoms. These findings highlight the potential value of incorporating personality traits into the understanding of symptom networks among college students.

## Figures and Tables

**Table 1:** Descriptive statistics and between-cohort comparisons of study variables.

Node	Symptom	T1 M (SD)	T1 Sk (Kt)	T2 M (SD)	T2 Sk (Kt)	*p*-Value	Cohen’s *d*
SOM	Somatization	1.368 (0.444)	1.900 (4.502)	1.437 (0.530)	1.759 (3.500)	<0.001	0.145
OC	Obsessive–compulsive	2.004 (0.611)	0.669 (0.253)	2.020 (0.689)	0.672 (0.115)	0.009	0.025
IS	Interpersonal sensitivity	1.802 (0.646)	0.964 (0.698)	1.783 (0.692)	1.024 (0.720)	0.001	−0.029
DEP	Depression	1.606 (0.601)	1.368 (1.875)	1.686 (0.676)	1.254 (1.314)	<0.001	0.126
ANX	Anxiety	1.601 (0.564)	1.470 (2.493)	1.599 (0.617)	1.446 (2.153)	0.702	−0.004
HOS	Hostility	1.513 (0.548)	1.671 (3.368)	1.538 (0.609)	1.718 (3.389)	<0.001	0.043
PHOB	Phobic anxiety	1.462 (0.515)	1.621 (3.063)	1.534 (0.600)	1.508 (2.397)	<0.001	0.131
PAR	Paranoia	1.571 (0.546)	1.340 (2.128)	1.573 (0.608)	1.384 (1.941)	0.739	0.003
PSY	Psychoticism	1.547 (0.508)	1.386 (2.255)	1.532 (0.572)	1.484 (2.367)	0.002	−0.029
-	Total score	133.782 (39.970)	1.254 (1.730)	136.118 (46.101)	1.244 (1.495)	<0.001	0.055
E	Extraversion	54.688 (11.019)	−0.444 (−0.297)	51.940 (11.895)	−0.214 (−0.763)	<0.001	−0.242
N	Neuroticism	49.672 (12.668)	−0.025 (−0.704)	50.282 (13.457)	−0.039 (−0.857)	<0.001	0.047
P	Psychoticism	44.311 (8.503)	0.768 (1.925)	45.701 (9.151)	0.900 (1.446)	<0.001	0.159

Note: M = Mean, SD = Standard Deviation, Sk = Skewness, Kt = Kurtosis. T1 refers to the pre-pandemic period, and T2 refers to the post-pandemic period. SCL-90 subscale scores are item-average scores; total score is the sum of all items. EPQ scores are T-scores; *p*-values and Cohen’s *d* are based on independent-samples t-tests comparing T1 and T2. All *p*-values were adjusted for multiple comparisons using the Holm–Bonferroni correction.

**Table 2:** Demographic characteristics of the samples.

Variables	Total n = 51,305		T1 n = 31,726		T2 n = 19,579		*p*-Value	Cramér’s *V*
	n	%	n	%	n	%		
Gender							<0.001	0.033
Male	14,605	28.47	8676	27.35	5929	30.28		
Female	36,606	71.35	23,049	72.65	13,557	69.24		
Hometown							<0.001	0.068
City	23,022	44.87	15,085	47.55	7937	40.54		
Rural areas	28,253	55.07	16,641	52.45	11,612	59.31		
Only child							<0.001	0.167
Yes	21,197	41.32	15,162	47.79	6035	30.82		
No	30,078	58.63	16,564	52.21	13,514	69.02		
Divorced family							<0.001	0.016
Yes	7458	14.54	4474	14.10	2984	15.24		
No	43,817	85.40	27,252	85.90	16,565	84.60		
Foster care experience							<0.001	0.070
Yes	5275	10.28	3790	11.95	1485	7.58		
No	46,000	89.66	27,936	88.05	18,064	92.26		
Left-behind experience							<0.001	0.025
Yes	18,449	35.96	11,711	36.91	6738	34.41		
No	32,826	63.98	20,015	63.09	12,811	65.43		
Childhood trauma							<0.001	0.068
Yes	9278	18.08	6393	20.15	2885	14.74		
No	41,997	81.86	25,333	79.85	16,664	85.11		

Note: Due to missing demographic information, the sample size varied slightly across variables. The number of missing values was 94 for gender and 30 each for hometown, only-child status, divorced family, foster care experience, left-behind experience, and childhood trauma. T1 refers to the pre-pandemic period, and T2 refers to the post-pandemic period. Group differences in categorical variables were examined using χ^2^ tests. All *p*-values were adjusted for multiple comparisons using the Holm–Bonferroni correction. Cramér’s *V* is reported as the effect size.

## Data Availability

The data are not publicly available due to privacy and ethical restrictions. De-identified data may be available from the corresponding author upon reasonable request, subject to the approval of the school’s administration and the signing of a data confidentiality agreement.

## Supplementary Materials

The following supporting information can be downloaded at: https://www.mdpi.com/article/10.3390/bs16071171/s1, Table S1: Descriptive statistics and gender differences in SCL-90 and EPQ scores; Table S2: Descriptive statistics and only-child differences in SCL-90 and EPQ scores; Table S3: Descriptive statistics and childhood trauma differences in SCL-90 and EPQ scores; Table S4: Edge weight matrix of the T1 period; Table S5: Edge weight matrix of the T2 period; Table S6: Expected impact index during T1 and T2 periods; Table S7: Node predictability values; Table S8: Significant p values and real differences in edge invariance; Table S9: Network comparison test results across academic years within T1 and T2 periods; Figure S1: Bootstrapped confidence intervals of edge weights; Figure S2: The stability estimation results of centrality indices.
