# Supplementary material for: Post-Pandemic Differences in Symptom Networks and Personality Associations Among College Students: A Repeated Cross-Sectional Study
Source: Behav Sci (Basel). 2026 Jul 11;16(7):1171. doi: 10.3390/bs16071171 (PMC13403485; doi:10.3390/bs16071171)
Supplement: Supplementary file 1 [file behavsci-16-01171-s001.zip › behavsci-4326451-supplementary.pdf]

Supplementary Materials

Supplementary Table S1. Descriptive statistics and gender differences in SCL-90 and EPQ scores

Supplementary Table S2. Descriptive statistics and only-child differences in SCL-90 and EPQ scores

Supplementary Table S3. Descriptive statistics and childhood trauma differences in SCL-90 and EPQ scores

Supplementary Table S4. Edge weight matrix of the T1 period

Supplementary Table S5. Edge weight matrix of the T2 period

Supplementary Table S6. Expected impact index during T1 and T2 periods

Supplementary Table S7. Node predictability values

Supplementary Table S8. Significant p values and real differences in edge invariance

Supplementary Table S9. Network comparison test results across academic years within T1 and T2 periods

Supplementary Figure S1. Bootstrapped confidence intervals of edge weights

Supplementary Figure S2. The stability estimation results of centrality indices

Supplementary Table S1. Descriptive statistics and gender differences in SCL-90 and EPQ scores

**Variables**	**G1_M**	**G2_M**	**G1_SD**	**G2_SD**	***p*_value**	**Cohen's *d***
SOM	1.364	1.406	0.464	0.485	<.000	-0.088
OC	1.962	2.029	0.656	0.635	<.000	-0.105
IS	1.761	1.807	0.666	0.662	<.000	-0.069
DEP	1.570	1.663	0.614	0.636	<.000	-0.147
ANX	1.544	1.622	0.571	0.588	<.000	-0.134
HOS	1.513	1.526	0.578	0.570	0.272	-0.022
PHOB	1.397	1.526	0.511	0.560	<.000	-0.235
PAR	1.579	1.568	0.586	0.563	0.464	0.019
PSY	1.541	1.541	0.556	0.524	1.000	-0.002
E	52.156	54.236	11.820	11.227	<.000	-0.182
N	49.410	50.091	14.597	12.264	<.000	-0.053
P	44.730	44.875	7.399	9.272	0.498	-0.017

Note: M = Mean, SD = Standard Deviation. G1 = Male group, G2 = Female group. SCL-90 subscale scores are item-average scores. EPQ scores are T-scores; *p*-values and Cohen's *d* are based on independent-samples t-tests. All *p*-values were adjusted for multiple comparisons using the Holm-Bonferroni correction.

Supplementary Table S2. Descriptive statistics and only-child differences in SCL-90 and EPQ scores

Variables	G1_M	G2_M	G1_SD	G2_SD	*p*_value	Cohen's *d*
SOM	1.377	1.407	0.468	0.487	<.000	-0.064
OC	1.986	2.027	0.637	0.645	<.000	-0.064
IS	1.769	1.813	0.656	0.669	<.000	-0.065
DEP	1.611	1.654	0.624	0.637	<.000	-0.068
ANX	1.582	1.613	0.578	0.589	<.000	-0.053
HOS	1.528	1.519	0.583	0.565	0.499	0.016
PHOB	1.451	1.516	0.525	0.566	<.000	-0.120
PAR	1.577	1.568	0.577	0.566	0.499	0.017
PSY	1.528	1.552	0.525	0.539	<.000	-0.045
E	54.695	52.897	11.261	11.506	<.000	0.158
N	49.914	49.897	12.951	12.996	1.000	0.001
P	44.787	44.879	8.769	8.791	0.967	-0.010

Note: M = Mean, SD = Standard Deviation. G1 = Only child group, G2 = Non-only child group. SCL-90 subscale scores are item-average scores. EPQ scores are T-scores; *p*-values and Cohen's *d* are based on independent-samples t-tests. All *p*-values were adjusted for multiple comparisons using the Holm-Bonferroni correction.

Supplementary Table S3. Descriptive statistics and childhood trauma differences in SCL-90 and EPQ scores

Variables	G1_M	G2_M	G1_SD	G2_SD	*p*_value	Cohen's *d*
SOM	1.631	1.342	0.601	0.431	<.000	0.619
OC	2.348	1.935	0.687	0.607	<.000	0.663
IS	2.207	1.704	0.742	0.609	<.000	0.792
DEP	2.046	1.546	0.744	0.565	<.000	0.831
ANX	1.945	1.524	0.707	0.524	<.000	0.749
HOS	1.819	1.458	0.71	0.515	<.000	0.650
PHOB	1.729	1.436	0.654	0.51	<.000	0.544
PAR	1.909	1.497	0.677	0.515	<.000	0.752
PSY	1.891	1.465	0.646	0.472	<.000	0.839
E	50.74	54.281	11.863	11.244	<.000	-0.312
N	57.086	48.317	11.706	12.706	<.000	0.700
P	48.388	44.058	9.751	8.352	<.000	0.502

Note: M = Mean, SD = Standard Deviation. G1 = Childhood trauma group, G2 = Non-childhood trauma group. SCL-90 subscale scores are item-average scores. EPQ scores are T-scores; *p*-values and Cohen's *d* are based on independent-samples t-tests. All *p*-values were adjusted for multiple comparisons using the Holm-Bonferroni correction.

Supplementary Table S4. Edge weight matrix of the T1 period

	SOM	OC	IS	DEP	ANX	HOS	PHOB	PAR	PSY	E	N	P
**SOM**	0.000	0.043	-0.087	0.074	0.293	0.130	0.078	0.018	0.096	0.057	0.000	0.034
**OC**	0.043	0.000	0.233	0.164	0.132	0.044	0.049	0.053	0.087	0.000	0.040	-0.098
**IS**	-0.087	0.233	0.000	0.190	0.110	0.072	0.169	0.235	0.134	-0.158	0.109	0.000
**DEP**	0.074	0.164	0.190	0.000	0.247	0.074	0.047	0.030	0.193	-0.141	0.159	0.043
**ANX**	0.293	0.132	0.110	0.247	0.000	0.084	0.198	0.056	0.174	0.000	0.065	-0.064
**HOS**	0.130	0.044	0.072	0.074	0.084	0.000	0.027	0.229	0.036	0.128	0.092	0.151
**PHOB**	0.078	0.049	0.169	0.047	0.198	0.027	0.000	0.000	0.008	-0.057	0.000	-0.022
**PAR**	0.018	0.053	0.235	0.030	0.056	0.229	0.000	0.000	0.236	0.062	-0.032	0.118
**PSY**	0.096	0.087	0.134	0.193	0.174	0.036	0.008	0.236	0.000	0.000	0.000	0.029
**E**	0.057	0.000	-0.158	-0.141	0.000	0.128	-0.057	0.062	0.000	0.000	-0.032	0.000
**N**	0.000	0.040	0.109	0.159	0.065	0.092	0.000	-0.032	0.000	-0.032	0.000	0.184
**P**	0.034	-0.098	0.000	0.043	-0.064	0.151	-0.022	0.118	0.029	0.000	0.184	0.000

Supplementary Table S5. Edge weight matrix of the T2 period

	SOM	OC	IS	DEP	ANX	HOS	PHOB	PAR	PSY	E	N	P
**SOM**	0.000	0.032	-0.096	0.060	0.329	0.127	0.063	0.051	0.093	0.063	0.000	0.055
**OC**	0.032	0.000	0.243	0.185	0.131	0.015	0.059	0.063	0.040	0.000	0.089	-0.125
**IS**	-0.096	0.243	0.000	0.200	0.088	0.074	0.199	0.228	0.150	-0.112	0.101	-0.011
**DEP**	0.060	0.185	0.200	0.000	0.252	0.087	0.058	0.029	0.187	-0.131	0.132	0.052
**ANX**	0.329	0.131	0.088	0.252	0.000	0.089	0.174	0.047	0.185	0.000	0.049	-0.061
**HOS**	0.127	0.015	0.074	0.087	0.089	0.000	0.035	0.221	0.054	0.113	0.066	0.160
**PHOB**	0.063	0.059	0.199	0.058	0.174	0.035	0.000	0.000	0.000	-0.082	0.000	-0.038
**PAR**	0.051	0.063	0.228	0.029	0.047	0.221	0.000	0.000	0.269	0.060	-0.018	0.113
**PSY**	0.093	0.040	0.150	0.187	0.185	0.054	0.000	0.269	0.000	0.000	0.000	0.028
**E**	0.063	0.000	-0.112	-0.131	0.000	0.113	-0.082	0.060	0.000	0.000	0.000	0.000
**N**	0.000	0.089	0.101	0.132	0.049	0.066	0.000	-0.018	0.000	0.000	0.000	0.136
**P**	0.055	-0.125	-0.011	0.052	-0.061	0.160	-0.038	0.113	0.028	0.000	0.136	0.000

Supplementary Table S6. Expected influence during T1 and T2 periods

Node	EI(T1)	EI(T2)	*p*-value	Test statistic E
**SOM**	0.736	0.775	0.178	0.039
**OC**	0.746	0.733	0.872	-0.013
**IS**	1.007	1.065	**0.007**	0.058
**DEP**	1.080	1.112	0.470	0.032
**ANX**	1.296	1.283	0.872	-0.013
**HOS**	1.068	1.040	0.535	-0.028
**PHOB**	0.497	0.467	0.518	0.030
**PAR**	1.004	1.063	**0.008**	0.059
**PSY**	0.994	1.006	0.872	0.012
**E**	-0.141	-0.090	0.206	0.051
**N**	0.584	0.555	0.535	-0.029
**P**	0.376	0.309	0.002	-0.067

Note: All reported values represent raw estimates; no standardization was applied. *p*-values were adjusted using the Holm–Bonferroni correction for multiple comparisons

Supplementary Table S7. Node predictability values

Node	R^2(T1)	R^2(T2)
**SOM**	0.588	0.665
**OC**	0.702	0.749
**IS**	0.807	0.843
**DEP**	0.815	0.851
**ANX**	0.821	0.854
**HOS**	0.634	0.691
**PHOB**	0.558	0.625
**PAR**	0.705	0.773
**PSY**	0.758	0.810
**E**	0.195	0.160
**N**	0.482	0.481
**P**	0.235	0.228

Supplementary Table S8. Significant *p*-values and real differences in edge invariance

**Node1**	**Node2**	***p*-value**	**Test statistic E**
OC	PSY	0.013	0.047
IS	E	0.013	0.045
OC	N	0.013	0.050
E	N	0.013	0.038
E	P	0.013	0.029
N	P	0.013	0.048

Note: *p*-values were adjusted using the Holm–Bonferroni correction for multiple comparisons.

Supplementary Table S9. Network comparison test results across academic years within T1 and T2 periods

Comparison	Network structure *p*	ΔM	Global strength *p*	ΔS
**2017 vs 2018**	**0.014**	0.058	0.935	0.006
**2017 vs 2019**	0.195	0.045	0.369	0.064
**2018 vs 2019**	**0.014**	0.059	0.303	0.070
**2023 vs 2024**	**0.020**	0.060	**0.018**	0.186

Note: *p*-values were adjusted using the Holm–Bonferroni correction for multiple comparisons

Supplementary Figure S1. Bootstrapped confidence intervals of edge weights

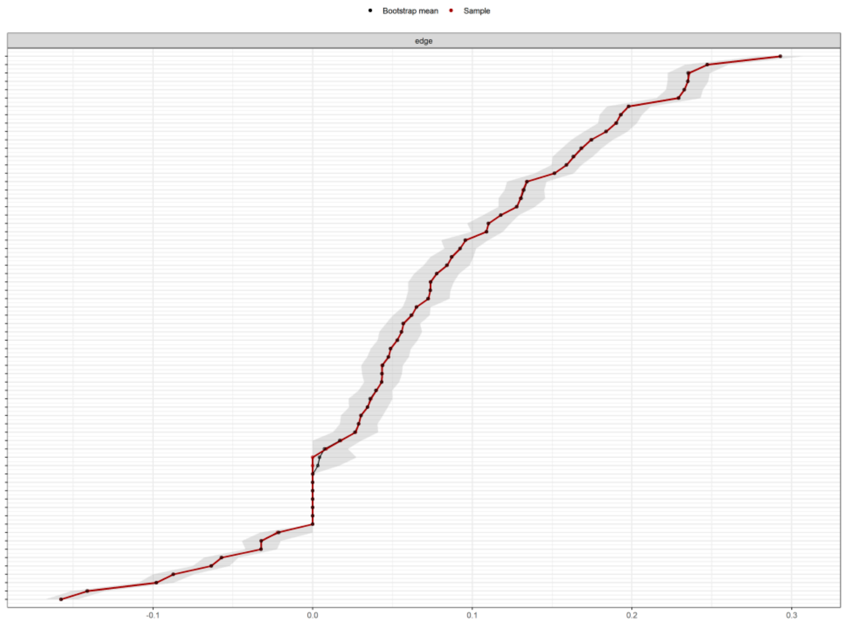

(a)T1 period

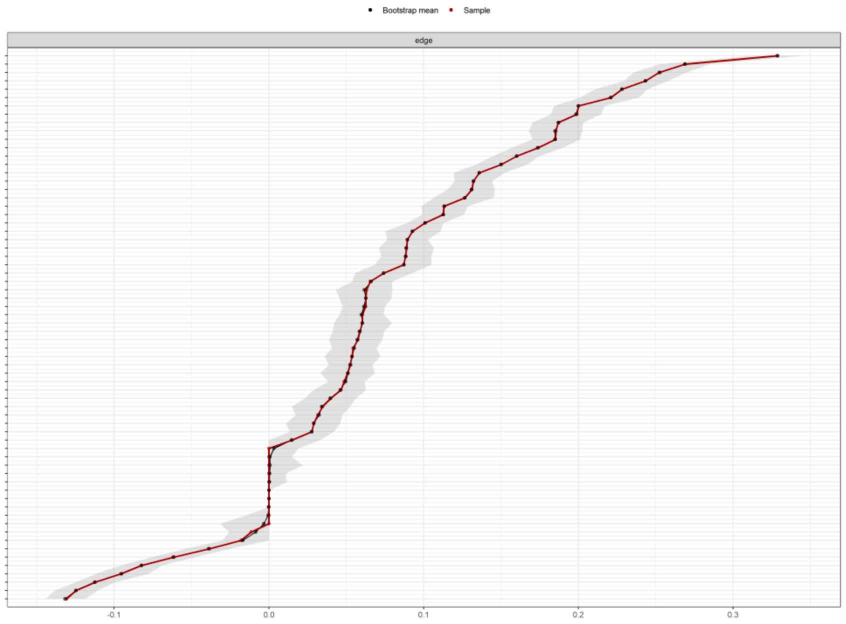

(b) T2 period

Note: The red dots represent the weight values of the samples, connected into lines, arranged in descending order from large to small. The black dots represent the processed weight values. The gray area represents the 95% confidence interval of the edge weight. The narrower the area, the higher the stability of the weight.

Supplementary Figure S2. The stability estimation results of centrality indices

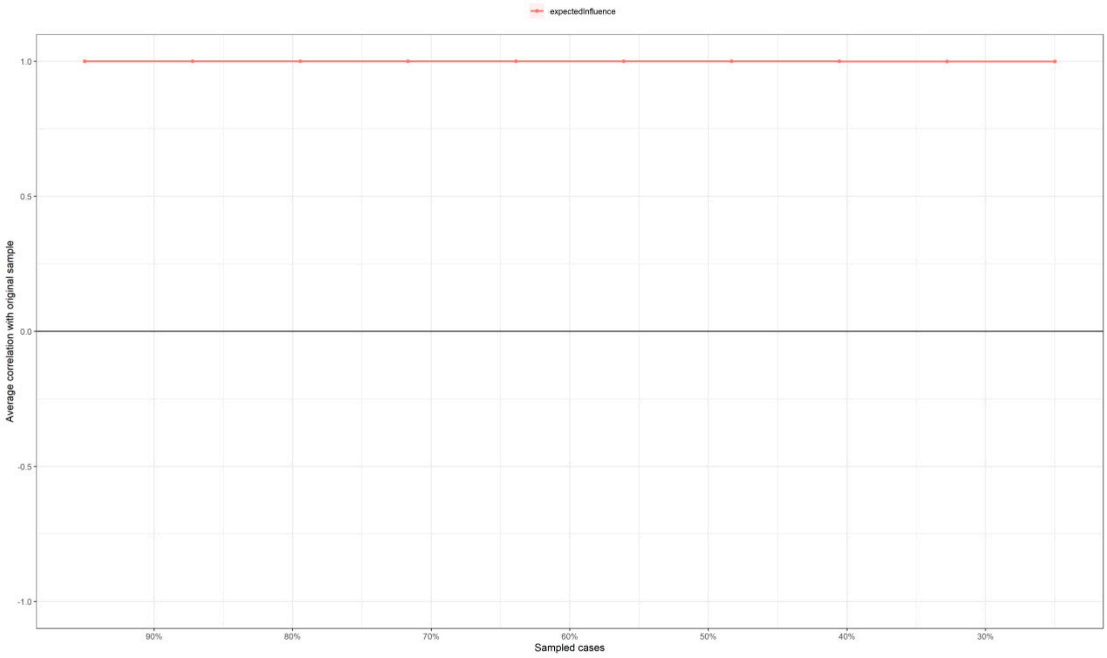

(a) T1 period

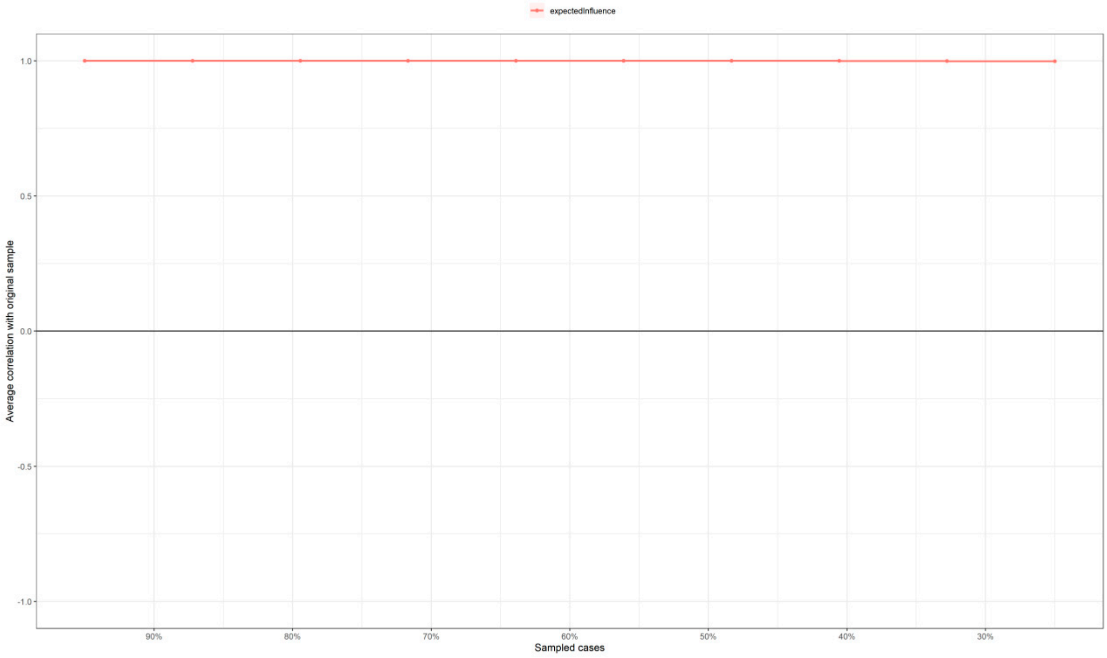

(b) T2 period
